# Supplementary material for: Social Outbreak in Chile, and Its Association with the Effects Biological, Psychological, Social, and Quality of Life
Source: Int J Environ Res Public Health. 2023 Nov 22;20(23):7096. doi: 10.3390/ijerph20237096 (PMC10706229; doi:10.3390/ijerph20237096)
Supplement: Supplementary file 1 [file ijerph-20-07096-s001.zip › ijerph-2581485-supplementary.pdf]

Supplementary Materials

Supplementary Figure S1. Frequency effect

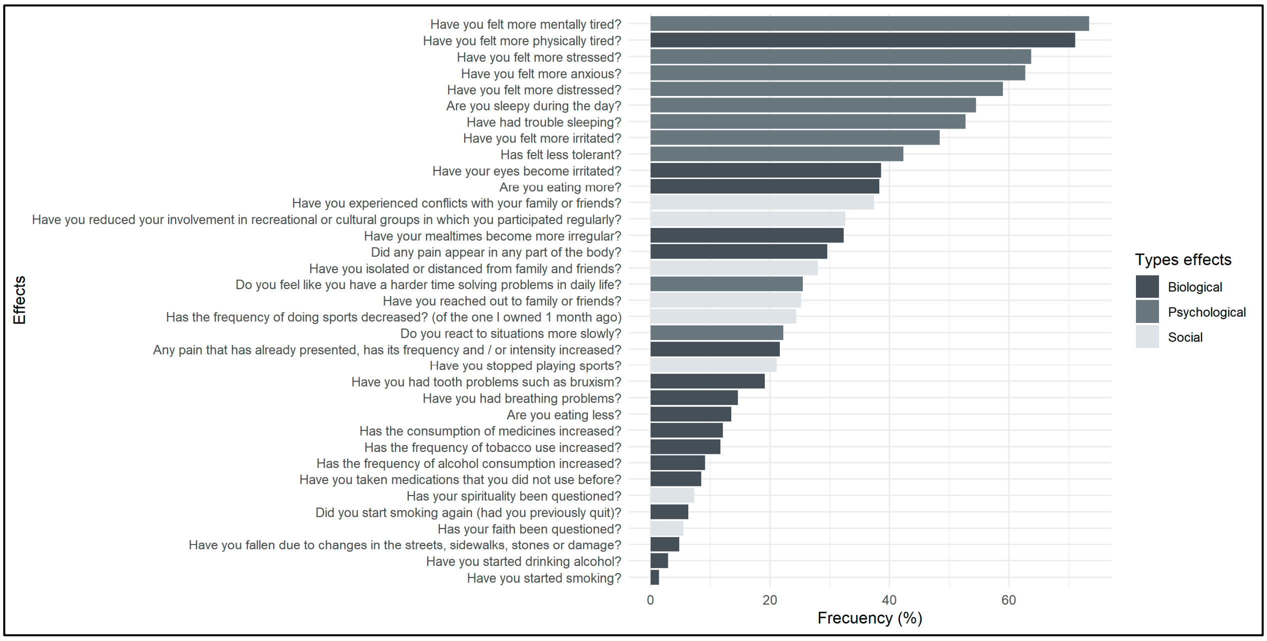

Data are presented in percentage.

Supplementary Table S1. Association biological effects and exposition

Symptomatology (%)	Overall	No exposition	Medium exposition	Higher exposition	*p*-Value
Have you felt more physically tired? (Yes)	1884 (71.1)	217 (11.5)	1163 (61.7)	504 (26.8)	<0.001
Have your eyes become irritated? (Yes)	1023 (38.6)	81 (7.9)	629 (61.5)	313 (30.6)	<0.001
Are you eating more? (Yes)	1016 (38.3)	135 (13.3)	614 (60.4)	267 (26.3)	0.022
Have your mealtimes become more irregular? (Yes)	856 (32.3)	84 (9.8)	520 (60.7)	252 (29.5)	<0.001
Did any pain appear in any part of the body? (Yes)	784 (29.6)	82 (10.5)	466 (59.4)	236 (30.1)	<0.001
Any pain that has already presented, has its frequency and / or intensity increased? (Yes)	573 (21.6)	66 (11.5)	341 (59.5)	166 (29.0)	0.001
Have you had tooth problems such as bruxism? (Yes)	506 (19.1)	57 (11.3)	291 (57.5)	158 (31.2)	<0.001
Have you had breathing problems? (Yes)	388 (14.6)	45 (11.6)	209 (53.9)	134 (34.5)	<0.001
Are you eating less? (Yes)	357 (13.5)	23 (6.4)	228 (63.9)	106 (29.7)	<0.001

Has the consumption of medicines increased? (Yes)	322 (12.1)	32 (9.9)	173 (53.7)	117 (36.4)	<0.001
Has the frequency of tobacco use increased? (Yes)	309 (11.7)	33 (10.7)	184 (59.5)	92 (29.8)	0.008
Has the frequency of alcohol consumption increased? (Yes)	241 (9.1)	19 (7.9)	153 (63.5)	69 (28.6)	0.004
Have you taken medications that you did not use before? (Yes)	225 (8.5)	29 (12.9)	111 (49.3)	85 (37.8)	<0.001
Did you start smoking again (had you previously quit)? (Yes)	166 (6.3)	18 (10.8)	98 (59.0)	50 (30.2)	0.076
Have you fallen due to changes in the streets, sidewalks, stones or damage? (Yes)	128 (4.8)	10 (7.8)	63 (49.2)	55 (43.0)	<0.001
Have you started drinking alcohol? (Yes)	76 (2.9)	6 (7.9)	47 (61.8)	23 (30.3)	0.146
Have you started smoking? (Yes)	38 (1.4)	5 (13.2)	21 (55.3)	12 (31.5)	0.496

Supplementary Table S2. Association psychological effects and exposition

**Symptomatology (%)**	**Overall**	**No exposition**	**Medium exposition**	**Higher exposition**	***p*-Value**
Have you felt more mentally tired? (Yes)	1946 (73.4)	230 (11.8)	1206 (62.0)	510 (26.2)	<0.001
Have you felt more stressed? (Yes)	1690 (63.7)	195 (11.6)	1040 (61.5)	455 (26.9)	<0.001
Have you felt more anxious? (Yes)	1662 (62.7)	205 (12.3)	1022 (61.5)	435 (26.2)	<0.001
Have you felt more distressed? (Yes)	1564 (59.0)	212 (13.6)	941 (60.2)	411 (26.2)	<0.001
Are you sleepy during the day? (Yes)	1445 (54.5)	171 (11.8)	882 (61.0)	392 (27.2)	<0.001
Have had trouble sleeping? (Yes)	1396 (52.7)	173 (12.4)	849 (60.8)	374 (26.8)	<0.001
Have you felt more irritated? (Yes)	1282 (48.4)	152 (11.9)	784 (61.1)	346 (27.0)	<0.001
Has felt less tolerant? (Yes)	1122 (42.3)	142 (12.7)	668 (59.5)	312 (27.8)	<0.001
Do you feel like you have a harder time solving problems in daily life? (Yes)	675 (25.5)	88 (13.0)	384 (56.9)	203 (30.1)	<0.001
Do you react to situations more slowly? (Yes)	589 (22.2)	57 (9.7)	361 (61.3)	171 (29.0)	<0.001

Supplementary Table S3. Association social effects and exposition

**Symptomatology (%)**	**Overall**	**No exposition**	**Medium exposition**	**Higher exposition**	***p*-Value**
Have you experienced conflicts with your family or friends? (Yes)	991 (37.4)	139 (14.0)	599 (60.4)	253 (25.6)	0.163
Have you reduced your involvement in recreational or cultural groups in which you participated regularly? (Yes)	865 (32.6)	109 (12.6)	502 (58.0)	254 (29.4)	<0.001

Have you isolated or distanced from family and friends? (Yes)	742 (28.0)	99 (13.3)	421 (56.7)	222 (30.0)	<0.001
Have you reached out to family or friends? (Yes)	669 (25.2)	69 (10.3)	431 (64.4)	169 (25.3)	0.002
Has the frequency of doing sports decreased? (of the one I owned 1 month ago) (Yes)	647 (24.4)	72 (11.1)	395 (61.1)	180 (27.8)	0.001
Have you stopped playing sports? (Yes)	560 (21.1)	53 (9.5)	341 (60.9)	166 (29.6)	<0.001
Has your spirituality been questioned? (Yes)	194 (7.3)	26 (13.4)	116 (59.8)	52 (26.8)	0.521
Has your faith been questioned? (Yes)	147 (5.5)	27 (18.4)	78 (53.1)	42 (28.5)	0.068

Supplementary Table S4. Characteristics by effect in QoL

	Overall	Not affected at all / little	Somewhat	To a great extent / extremely	*p*-Value
**n**	2651	790 (29.8)	1064 (40.1)	797 (30.1)	
**Sex (%)**					
Man	774 (29.2)	293 (37.9)	294 (38.0)	187 (24.1)	<0.001
Woman	1877 (70.8)	497 (26.5)	770 (41.0)	610 (32.5)	
**Age (mean (SD))**	35.22 (11.30)	33.73 (10.70)	34.79 (10.86)	37.27 (12.16)	<0.001
**Education (%)**					
Up to high school	96 (3.6)	26 (27.1)	33 (34.4)	37 (38.5)	0.181
Incomplete College	108 (4.1)	33 (30.6)	34 (31.5)	41 (37.9)	
College / technical Degree	2447 (92.3)	731 (29.9)	997 (40.7)	719 (29.4)	
**Occupation (%)**					
Inactive	155 (5.8)	37 (23.9)	54 (34.8)	64 (41.3)	0.001
Employer	157 (5.9)	33 (21.0)	57 (36.3)	67 (42.7)	
Student	315 (11.9)	89 (28.3)	133 (42.2)	93 (29.5)	
Worker	1462 (55.1)	446 (30.5)	596 (40.8)	420 (28.7)	
Independent	562 (21.3)	185 (32.9)	224 (39.9)	153 (27.2)	
**Zone (%)**					
North	229 (8.6)	59 (25.8)	82 (35.8)	88 (38.4)	<0.001
Centre	716 (27.0)	159 (22.2)	251 (35.1)	306 (42.7)	
South	280 (10.6)	49 (17.5)	127 (45.4)	104 (37.1)	
Metropolitan region	1426 (53.8)	530 (37.2)	330 (23.1)	566 (39.7)	

Supplementary Table S5: Association Place exposition and effect in QoL

	Overall	Not affected at all / little	Somewhat	To a great extent / Extremely	OR 95% CI	*p*-Value
**Closeness disturbs home (%)**						
At the disturb place	228 (8.6)	27 (11.8)	54 (23.7)	147 (64.5)	5.72 (3.54; 9.23)	<0.001
2-3 block from disturbs.	491 (18.5)	109 (22.2)	197 (40.1)	185 (37.7)	2.85 (2.04; 3.98)	<0.001
4-5 blocks from disturbs	913 (34.4)	266 (29.2)	401 (43.9)	246 (26.9)	1.95 (1.45; 2.61)	<0.001
Far away from disturbs.	759 (28.6)	277 (36.5)	315 (41.5)	167 (22.0)	1.41 (1.05; 1.90)	0.022
In an area where there were no disturbances	260 (9.9)	111 (42.7)	97 (37.3)	52 (20.0)	1.00 (Ref.)	
**Closeness disturbs work (%)**						
At the disturb place	394 (14.9)	91 (23.1)	167 (42.4)	136 (34.5)	2.92 (2.02; 4.22)	<0.001
2-3 block from disturbs						

	662 (25.0)	171 (25.8)	272 (41.1)	219 (33.1)	2.40 (1.72; 3.34)	<0.001
4-5 blocks from disturbs	665 (25.1)	187 (28.1)	286 (43.0)	192 (28.9)	2.10 (1.51; 2.91)	<0.001
Far away from disturbs.	591 (22.3)	206 (34.9)	239 (40.4)	146 (24.7)	1.56 (1.13; 2.17)	0.008
In an area where there were no disturbances	209 (7.9)	94 (45.0)	61 (29.2)	54 (25.8)	1.00 (Ref.)	
No work, no study	130 (4.8)	41 (31.5)	39 (30.0)	50 (38.5)	1.01 (0.60; 1.69)	0.975
**Closeness disturbs shop (%)**						
At the disturb place	856 (32.3)	213 (24.9)	332 (38.8)	311 (36.3)	3.65 (2.38; 5.61)	<0.001
2-3 block from disturbs	720 (27.2)	208 (28.9)	309 (42.9)	203 (28.2)	2.87 (1.87; 4.42)	<0.001
4-5 blocks from disturbs	551 (20.8)	171 (31.0)	228 (41.4)	152 (27.6)	2.28 (1.47; 3.53)	<0.001
Far away from disturbs	420 (15.8)	146 (34.7)	165 (39.3)	109 (26.0)	1.92 (1.23; 3.01)	0.004
In an area where there were no disturbances	104 (3.9)	52 (50.0)	30 (28.8)	22 (21.2)	1.00 (Ref.)	

Data presented as frequency and percentage, and Odds Ratio (OR) with their 95% Confidence Interval (CI). Model adjusted by sex, age, region, education and occupation.

Supplementary Figure S2. Frequency disturbs (A) and resources for information (B).

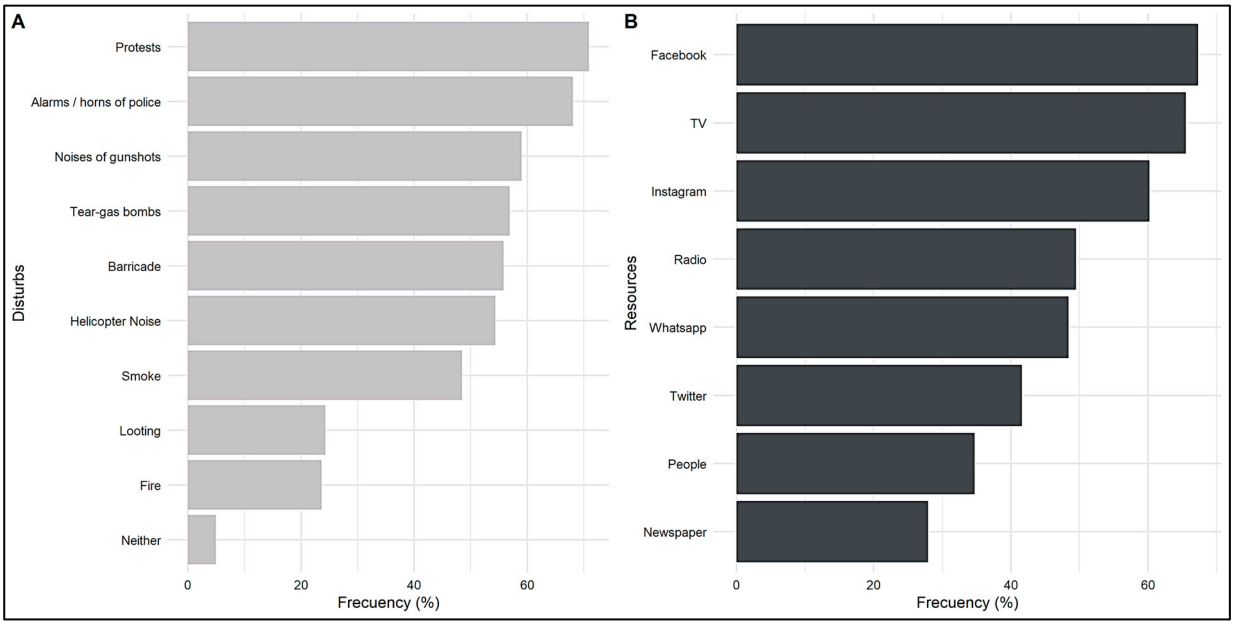

Data are presented in percentage.
